# Pixel-Wise Interstitial Lung Disease Interval Change Analysis: A Quantitative Evaluation Method for Chest Radiographs Using Weakly Supervised Learning

**DOI:** 10.3390/bioengineering11060562

**Published:** 2024-06-02

**Authors:** Subin Park, Jong Hee Kim, Jung Han Woo, So Young Park, Yoon Ki Cha, Myung Jin Chung

**Affiliations:** 1 Department of Health Sciences es and Technology, SAIHST, Sungkyunkwan University, Seoul 06351, Republic of Korea; subinn.park@gmail.com (S.P.);; 2 Department of Radiology, Samsung Medical Center, Sungkyunkwan University School of Medicine, Seoul 0631, Republic of Korea; jonghk1101@naver.com (J.H.K.);; 3 Medical AI Research Center, Research Institute for Future Medicine, Samsung Medical Center, Seoul 06351, Republic of Korea

**Keywords:** interstitial lung disease, quantification, extent analysis, weakly supervised learning, image-to-image translation, interval change analysis

## Abstract

Interstitial lung disease (ILD) is characterized by progressive pathological changes that require timely and accurate diagnosis. The early detection and progression assessment of ILD are important for effective management. This study introduces a novel quantitative evaluation method utilizing chest radiographs to analyze pixel-wise changes in ILD. Using a weakly supervised learning framework, the approach incorporates the contrastive unpaired translation model and a newly developed ILD extent scoring algorithm for more precise and objective quantification of disease changes than conventional visual assessments. The ILD extent score calculated through this method demonstrated a classification accuracy of 92.98% between ILD and normal classes. Additionally, using an ILD follow-up dataset for interval change analysis, this method assessed disease progression with an accuracy of 85.29%. These findings validate the reliability of the ILD extent score as a tool for ILD monitoring. The results of this study suggest that the proposed quantitative method may improve the monitoring and management of ILD.

## 1. Introduction

Interstitial lung disease (ILD) comprises diffuse pulmonary parenchymal disorders, with fibrotic ILD leading to poor outcomes with progression [1]. The early detection of ILD disease progression is clinically important, given that antifibrotic drugs may have effects on the annual decline of forced vital capacity in patients with progressive pulmonary fibrosis (PPF) and idiopathic pulmonary fibrosis (IPF) [2,3].

Although ILD is reliably detected by high-resolution computed tomography (HRCT), a chest radiograph is still commonly used [4]. In contrast to HRCT, chest radiographs are available in almost all facilities and can be used quickly with minimal cost and negligible radiation [5]. However, the interpretation of chest radiographs can be challenging, even for experienced radiologists, and is prone to low interobserver agreement [6,7]. According to a previous study, chest radiographs miss nearly 30% of cases when used for screening of fibrotic ILD [4]. Thus, considerable effort has been devoted to developing deep learning–based computer-aided detection systems for the evaluation of ILD on chest radiographs. However, previous studies have focused mainly on the detection of ILD on chest radiographs (Table 1) [6,7,8,9,10]. Previous studies have shown that a deep learning algorithm may be superior to interpretation by radiologists, facilitating the detection of reticular opacity on chest radiographs in the early stages of ILD [6,7,8,9,10].

However, despite the importance of changes in chest radiographs in determining treatment timing and modifying treatment strategies, since previous studies have focused only on the detection of ILD in chest radiographs (CXR), the assessment of changes in chest radiographs has largely relied on subjective visual interpretation, which limits its ability to capture disease changes over time. Additionally, based on our knowledge quantitative assessments have been based on CT imaging [11]. Predicting therapeutic response and prognosis through quantitative assessment was carried out by using CT imaging by Lancaster et al. and Kim et al. [12,13].

**Figure 1:**
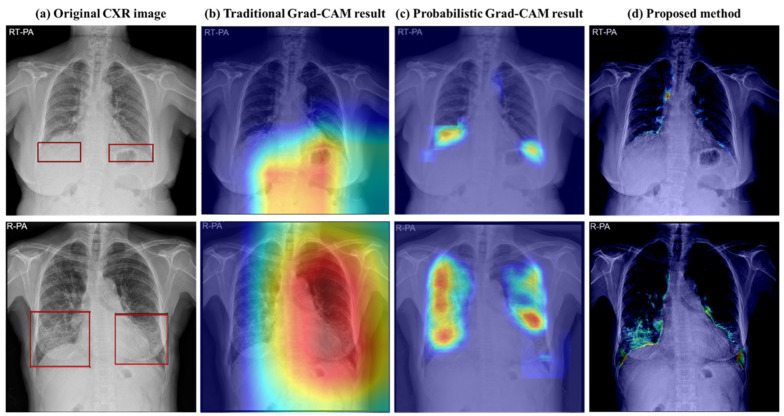
Results of Grad-CAM method and image-to-image translation method: (**a**) original CXR image: the original chest X-ray of a patient with ILD, and the red box indicates the abnormal area; (**b**) traditional Grad-CAM results: images with abnormal areas are highlighted using Grad-CAM; (**c**) probabilistic Grad-CAM results: images with probabilistic Grad-CAM were used for more detailed area analysis; (**d**) proposed method (image-to-image translation method) results: analyzing the images using the method proposed in this study, the ILD areas were accurately displayed in pixel units compared to Grad-CAM.

Thus, the quantitative assessment and visualization of changes in chest radiographs of ILD patients may be a noteworthy advance in disease management. However, ILD regions are widely distributed and have unclear boundaries; thus, accurate pixel-level labeling is complicated. Previous studies have used a gradient-weighted class activation map (Grad-CAM) [14] to detect disease locations when the exact site cannot be directly indicated [15,16,17]. Grad-CAM has also been applied to detect ILD areas, but they often appear more extensive than actual lesions and also include some normal areas expressed as probability values [8,9]. This lack of detail may hinder accurate measurement of the severity and progression of ILD (Figure 1b,c).

To improve this, recent research has focused on lesion area detection using image-to-image translation models, e.g., in studies by DeGrave et al. and Li et al. Although CycleGAN (cycle-consistent adversarial networks) [18] has been applied to detect abnormal regions in COVID-19, this approach has not yet been applied to ILD [19,20]. Therefore, in this study, ILD region detection was performed using the CUT (contrastive unpaired translation) model [21], an image-to-image translation model (Figure 1d). The CUT model was trained to translate images with ILD lesions into normal virtual images. By comparing the original ILD image to the virtual normal image, a pixel-wise abnormal area can be obtained [19,20]. We designed a new ILD extent scoring algorithm to quantitatively evaluate changes in the disease in the pixel-wise disease area. The final calculated extent score was verified using CXR follow-up data.

In other words, this study demonstrates that ILD disease areas in chest radiographs can be detected using an image-to-image translation model. The areas verified through the ILD extent scoring algorithm are presented in quantitative terms, allowing for the measurement and verification of changes in chest radiographs of ILD patients through calculated quantitative values. This approach may help clinicians determine and tailor management strategies for patients with ILD in the future.

The article is organized as follows: The Materials and Methods section describes the dataset used, the models implemented, and the methods for evaluation. The Results section presents the study’s results, including a performance comparison of various models for ILD classification and abnormal area detection. The Discussion section discusses the findings, compares them with related studies, and outlines the implications for clinical practice. Finally, the Conclusions section concludes the article with a summary of the contributions and potential directions for future research.

## 2. Materials and Methods

### 2.1. Study Design

The institutional review board (IRB) of Samsung Medical Center approved this retrospective study. The requirement for patient consent to use clinical data was waived by the IRB due to the retrospective study design (IRB file number: 2022-03-138). All patient-identifying information was removed from the images.

**Figure 2:**
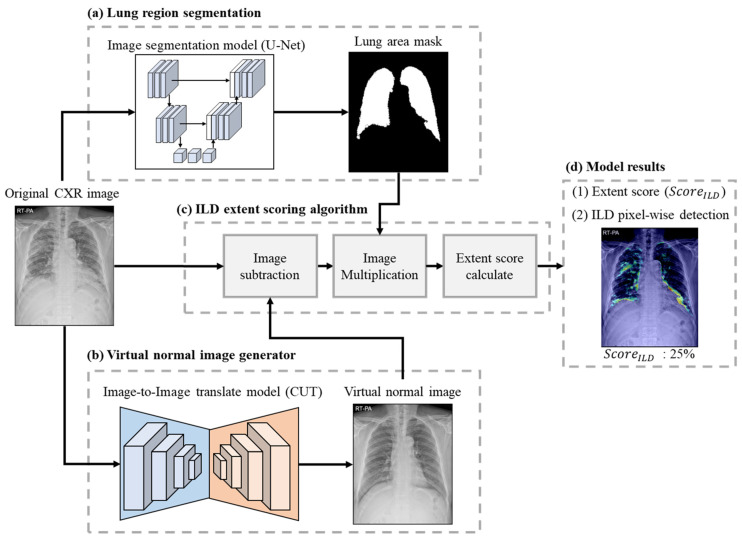
Proposed ILD extent score quantification method: (**a**) lung region segmentation: a binary mask was generated by segmenting the lung region of the original CXR; (**b**) virtual normal image generator: the original CXR image was used as input to generate a virtual normal image without abnormal areas; (**c**) ILD extent scoring algorithm: the ILD area was obtained using the original CXR image and the virtual normal image in (**b**), and the lung area and the ILD area were compared using the lung area mask in (**a**) to determine the extent score of the ILD ($Score_{ILD}$); (**d**) model results: the model visualizes the extent score and pixel-wise detection results of ILD.

This study presents a weakly supervised learning approach for quantifying ILD severity on CXR. The ILD quantitative evaluation method proposed in this study quantifies the disease area using the output of two neural network architectures: (a) lung area segmentation and (b) a virtual normal image generator (Figure 2a,b).

In this section, we describe the methodology used for collecting and processing the CXR dataset, implementing a neural network model for lung area segmentation (Figure 2a), implementing an image-to-image translation model for creating a virtual normal image (Figure 2b), and the ILD extent scoring algorithm (Figure 2c).

### 2.2. Datasets

A retrospective search of the surgical database of the center was conducted to identify patients with ILD, surgically confirmed by diagnostic pulmonary wedge resection performed between January 2012 and December 2023. The training dataset included patients between January 2016 and December 2018. For the evaluation dataset, between two and four follow-up chest radiographs from each patient with ILD were used, and the dataset included chest radiographs taken between January 2012 and December 2023.

**Figure 3:**
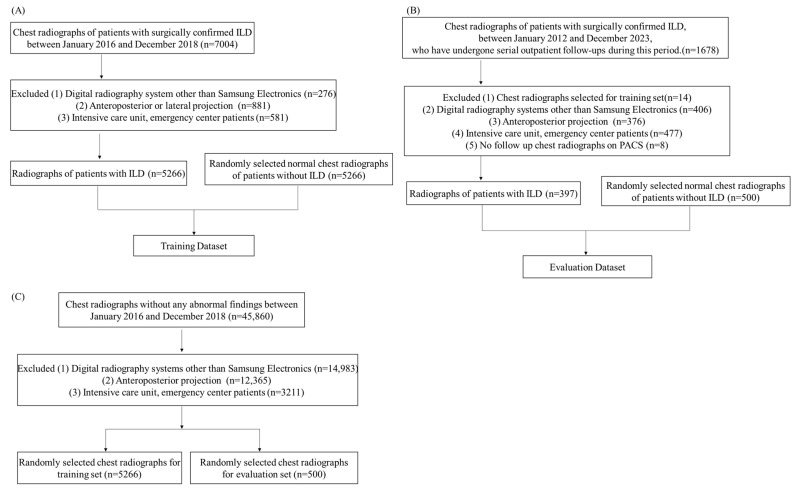
Flowchart showing patient selection: (**A**,**B**) patient selection for training and evaluation dataset; (**C**) patient selection for the control group (i.e., patients without ILD and with normal radiographs).

Chest radiographs taken with digital radiography systems other than Samsung Electronics were excluded. Furthermore, chest radiographs with anteroposterior and lateral projections were excluded. Lastly, chest radiographs of intensive care unit and emergency center patients were excluded. This is summarized in Figure 3 and Table 2.

From the collected image-to-image translation dataset, 100 lungs were annotated: 50 representing normal lung fields and 50 with manifestations of ILD. The annotated samples were split into training and validation sets, comprising 80% and 20% of the images, respectively, with equal distribution of normal and ILD cases in both subsets.

### 2.3. Image Acquisition

All radiographs were obtained with the patient in the erect position with posteroanterior projection using the following digital radiography systems: DGR-U6LN2A, DGR-C55J29, DGR-U3QN2D/KR (Samsung Electronics, Suwon, Republic of Korea).

### 2.4. Reference Standard

To establish the reference standard for the detection of abnormal lesions on chest radiographs, two radiologists (Myung Jin Chung, with 30 years, and Jong Hee Kim with 7 years of experience in radiology, Samsung Medical Center, Sungkyunkwan University School of Medicine, Seoul, Republic of Korea) in consensus outlined abnormalities on chest radiographs based on direct visual comparison and patient chest CT examinations. For all ILD patients, signs of fibrosis were confirmed using CT images (for some patients, the chest radiograph and CT images were taken on different days).

The radiologists classified chest radiograph changes into three categories based on a visual assessment of the bilateral percentage involvement of the changes in total lung parenchyma by reticular opacities, consolidation, and ground glass opacities: aggravation, improvement (changes involving more than one-third of each hemithorax), no change (changes involving less than one-third of each hemithorax).

### 2.5. Model Structure

#### 2.5.1. Lung Area Segmentation

The lung area is a reference value for quantifying the severity of ILD. In CXR images, lung area may vary depending on the shooting angle and location. Thus, for accurate quantification, the lung area must be set as a reference value. In this study, lung region segmentation was performed using the U-Net architecture [22], as shown in Figure 2a. U-Net is a widely used standard model in medical image analysis. The original CXR images were first resized to a resolution of 512 × 512 pixels^2^ and preprocessed via CLAHE [23]. This resolution balances efficiency and accuracy for detecting clear lung boundaries. U-Net learned how to distinguish between the lung area (area with a pixel value of 1) and the remaining area (area with a pixel value of 0) by inputting preprocessed images. U-Net was trained for 100 epochs for each experiment, with the initial learning weight set to 0, the batch size set to 2, and the Adam optimizer [24] with a learning rate of 1 × 10^−4.^ To prevent overfitting, the dropout rate [25] was set to 0.2, and early stopping was performed based on the validation loss.

#### 2.5.2. Virtual Normal Image Generator

The virtual normal image generator translated the CXR image of an ILD patient into a virtual normal dataset without abnormal areas, as shown in Figure 2b. This model uses CUT (contrastive unpaired translation) [21], an image-to-image translation model. The CUT model is a type of generative model designed to perform image-to-image translation tasks without paired training data. The network was trained to generate a ‘virtual normal’ image by mapping data containing disease areas to a ‘normal’ shape. The CUT model followed the architecture proposed in the original paper [21]. The original CXR image was resized to 1024 × 1024 resolution, maintaining the aspect ratio after min–max normalization; the remaining area was used as input after zero-padding. CUT was trained using the Adam optimizer with initial weights set to random distributions, a batch size of 2, and a learning rate of 0.0002 for 250 iterations per experiment [24].

#### 2.5.3. ILD Extent Scoring Algorithm

**Figure 4:**
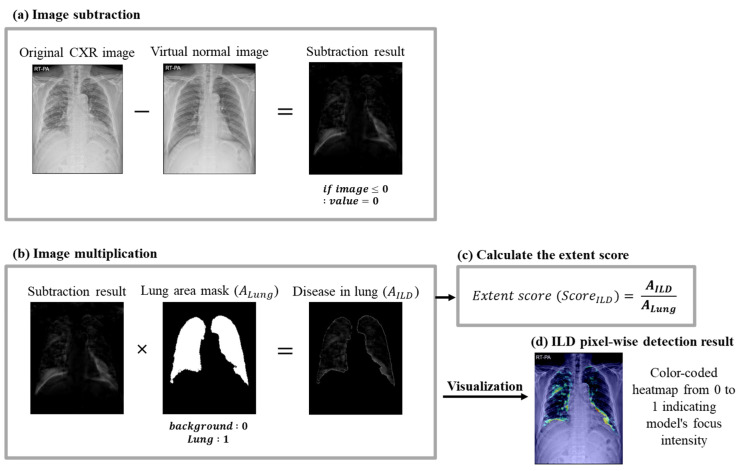
Multi-stage ILD extent scoring algorithm process for ILD quantification: (**a**) image subtraction: the original CXR image is subtracted from the virtual normal image generated by the virtual normal image generator to highlight abnormal areas indicative of ILD; (**b**) image multiplication: the lung mask area calculated in the lung area segmentation process is multiplied by the abnormal area calculated in (**a**) to determine the abnormal area in the lung; (**c**) the extent score is calculated by dividing the abnormal area by the lung area; (**d**) ILD pixel-wise detection result: a color-coded heatmap indicates disease severity and distribution, showing pixel-wise detection of ILD.

In the final step, the ILD extent score ($Score_{ILD}$) was calculated in a three-stage process, using outputs from the two neural networks as inputs, as shown in Figure 2c. Each stage is detailed in Figure 4. First, image subtraction was performed to discern the difference between the original CXR image and the virtual normal image (Figure 4a). The lung area mask was resized to 1024 × 1024 using bilinear interpolation to match the virtual normal image size. The subtraction result was multiplied by the lung area mask to determine the abnormal area within the lung region (Figure 4b). The extent score was derived by dividing the area of detected abnormality (the result of the image subtraction, $A_{ILD}$) by the lung area (the output from the lung area segmentation, $A_{Lung}$). An extent score near 0 indicates normalcy; a score approaching 1 signifies more severe disease.

### 2.6. Development Environment

The computing environment for development and testing used high-performance computing resources. All experiments were performed on a platform equipped with two NVIDIA V100 GPUs using PyTorch (version 1.4.0) in Python 3.6. Each model was trained until performance saturation was observed on the validation set, ensuring optimal learning without overfitting.

### 2.7. Model Evaluation

The evaluation method in this study was designed to test the accuracy of ILD quantification. This includes evaluating lung area segmentation, image-to-image translation, ILD classification, and ILD interval change classification over time.

The performance of lung area segmentation was assessed using the Dice score, a statistical tool that measures the overlap between manual annotations and model predictions.

The ability of the CUT model to generate virtual normal images was evaluated by analyzing the fidelity and quality of the synthesized images. To provide a comparative analysis, the performance of the CycleGAN model was also assessed. The CycleGAN model followed the architecture proposed in the original paper [18]. Both models were evaluated using the structural similarity index (SSIM) and peak signal-to-noise ratio (PSNR) by comparing the original CXR images with the virtual normal images.

For disease classification, images were labeled based on the presence of ILD (normal = 0, ILD = 1). An extent score ($Score_{ILD}$) of 0% indicates a normal classification; a $Score_{ILD}$ above 0% indicates the presence of ILD (normal: $Score_{ILD}=0\%$, ILD: $Score_{ILD}>$ 0%). To evaluate the classification performance of $Score_{ILD}$, we implemented and compared baseline classification models. Baseline classification models used as comparisons include VGG16 [26], ResNet-34 [27], EfficientNet-B0 [28], and vision transformer (ViT) [29]. We also compared GradCAM models (ResNet-34 and probabilistic Grad-CAM) [16]. The VGG16 model followed the architecture proposed in the original paper by leveraging pre-trained weights [26]. The ResNet-34 model was implemented using a pretrained ResNet-34 model that followed the architecture described in the original ResNet paper [27]. The EfficientNet model was implemented using the pretrained EfficientNet-B0 model according to the architecture described in [28]. The ViT model is based on the architecture proposed in the original ViT paper and was implemented using a pretrained ViT base model with a patch size of 16 and an input size of 224 [29]. The Grad-CAM model was implemented following the architecture described in [16]. Classification performance was analyzed using accuracy, precision, recall, and F1-score metrics.

The ability of the model to track disease progression or regression was evaluated using a follow-up dataset to assess changes in disease state (aggravation = 1, no change = NC = 2, improvement = 3). The classification was based on differences in the extent score between images, with a 5% change threshold set as the criterion for disease progression. Cases showing changes exceeding this threshold were classified accordingly (increases of more than 5% as aggravation, decreases of more than 5% as improvement); minimal changes were considered stable (no change).

## 3. Results

### 3.1. Lung Area Segmentation Performance

**Figure 5:**
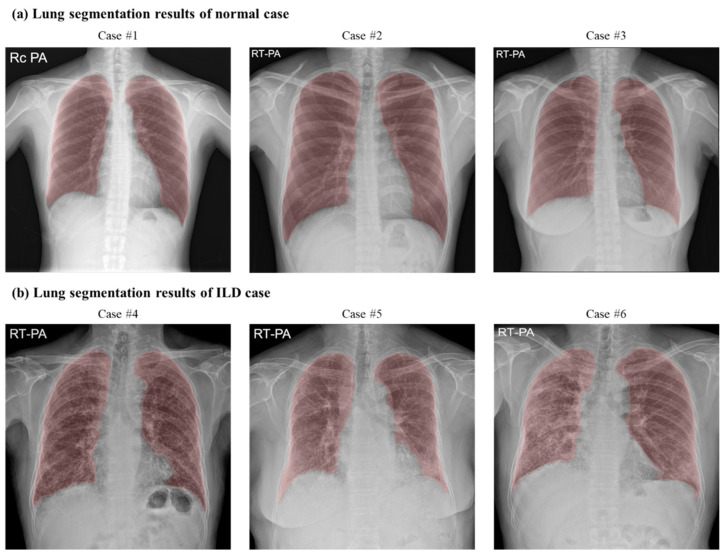
U-Net segmentation network results for normal and ILD datasets. The red region represents the output of the U-Net segmentation network: (**a**) lung segmentation results for the normal dataset; (**b**) lung segmentation results for the ILD dataset show that the network accurately delineates lung regions in the presence of ILD lesions. The results demonstrate the effectiveness of the network in accurately segmenting lung regions in both healthy and diseased lung tissue.

The lung area segmentation achieved a Dice score of 0.93, reflecting the high network performance across both ILD and normal datasets. Figure 5a,b display the segmentation results for each dataset. The network accurately segmented lung areas in both ILD and normal lungs, indicating reliable outcomes in the original CXR images.

### 3.2. Image-to-Image Translation Fidelity

**Figure 6:**
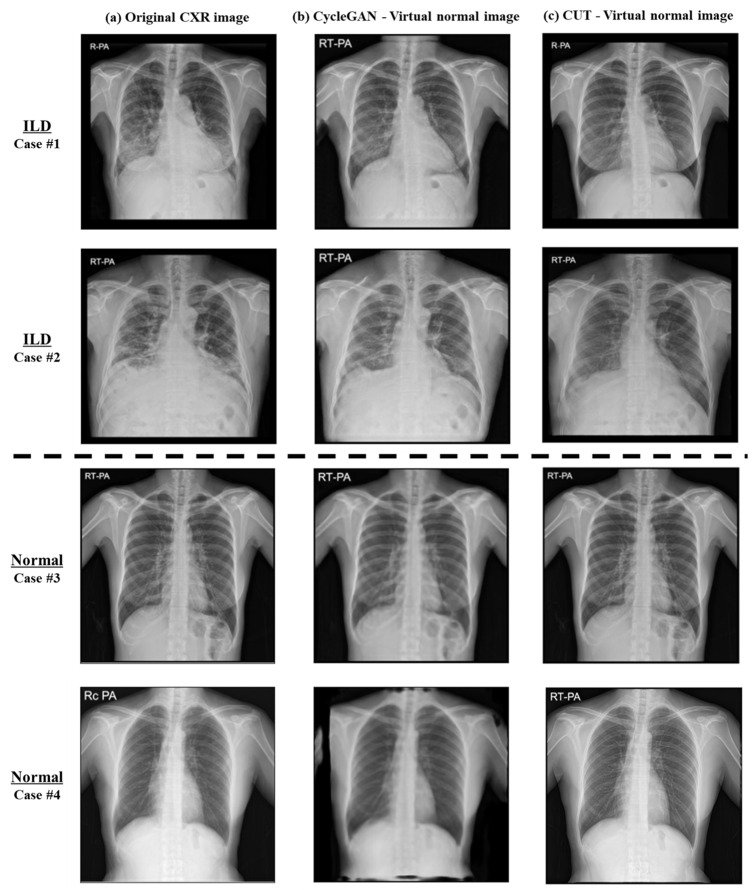
The visual results of translation models to CXR images: (**a**) original CXR image from the dataset; (**b**) virtual normal image generated by the CUT model; (**c**) virtual normal image generated by the CycleGAN model.

The ability of both the CycleGAN and CUT models to generate virtual normal images was evaluated with a focus on the fidelity and quality of the synthesized images. The performance was quantitatively assessed using SSIM and PSNR by comparing the original CXR images (Figure 6a) with the virtual normal images (Figure 6b,c). As shown in Table 3, for the normal dataset, the CUT model achieved an SSIM of 0.97 and a PSNR of 36.43, indicating near-perfect structural similarity to the original images. The CycleGAN model, on the other hand, achieved an SSIM of 0.88 and a PSNR of 23.68. In the ILD dataset, the CUT model achieved an SSIM of 0.90 and a PSNR of 26.61, while the CycleGAN model achieved an SSIM of 0.71 and a PSNR of 18.46. Although the SSIM and PSNR were lower for both models in the ILD dataset compared to the normal dataset due to the removal of abnormal areas in the lung region, the high score of 0.90 for the CUT model demonstrates the model’s effectiveness in preserving structural details of the skeletal structure.

Figure 6a shows the original CXR image, Figure 6b shows the virtual normal image generated by the CycleGAN model, and Figure 6c shows the virtual normal image generated by the CUT model. In Cases 1 and 2 with ILD areas, the virtual normal image of the CUT model effectively removed the abnormal area while preserving the overall skeletal structure. The CycleGAN model also removed abnormal areas, but its precision and structural integrity were lower than those of the CUT model. In Cases 3 and 4 (normal), there were no abnormalities in the chest radiograph, so the virtual normal images of both models were similar to the original CXR images, but the CUT model maintained higher fidelity.

### 3.3. ILD Classification Accuracy

**Figure 7:**
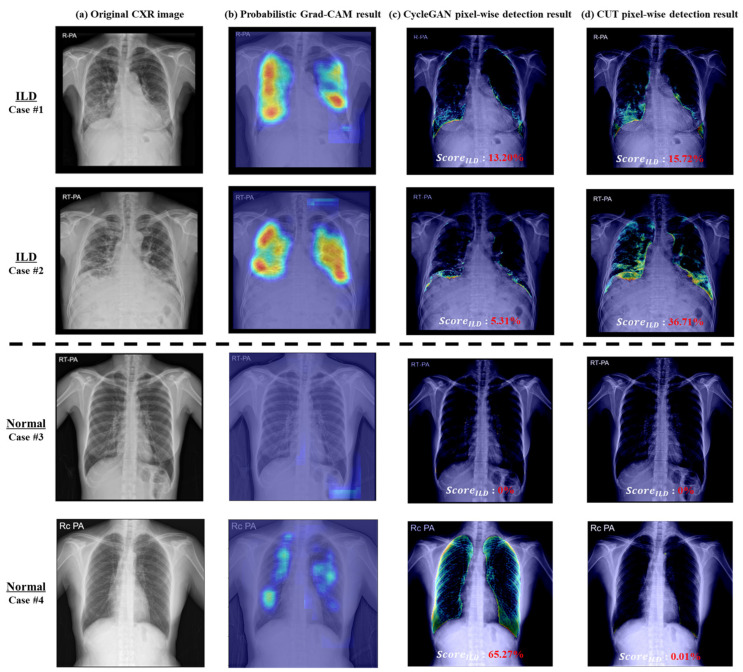
The visual results of abnormal area detection: (**a**) original CXR images from the datasets; (**b**) probabilistic Grad-CAM results, showing the detected abnormal areas; (**c**) CycleGAN with extent scoring algorithm results, showing the detected abnormal areas; (**d**) CUT with extent scoring algorithm results, showing the detected abnormal areas. In the case of ILD, the detected abnormal areas are highlighted, whereas in normal cases, the abnormal areas are not detected.

The extent scores ($Score_{ILD}$) of the proposed model were evaluated for classification performance between normal and ILD cases. As shown in Figure 7, in ILD cases (Cases 1 and 2), the extent score exceeded 0%; in normal cases (Cases 3 and 4), the extent score was 0%, consistent with the absence of disease.

For performance analysis, the proposed model was benchmarked against several baseline models, including VGG16, ResNet-34, EfficientNet-B0, ViT, and probabilistic Grad-CAM models. The results are shown in Table 4. The proposed method (CUT with ILD extent scoring algorithm) demonstrated high performance, with an accuracy of 92.98% and an F1-score of 95.68%. The probabilistic Grad-CAM method achieved an accuracy of 84.76% and an F1-score of 84.02%. The CycleGAN model, when applied with the extent scoring algorithm, achieved an accuracy of 81.20% and an F1-score of 76.96%. The performance of baseline classification models was also evaluated, showing that the VGG16 model achieved an accuracy of 68.65%, the ResNet-34 model achieved an accuracy of 92.11%, the EfficientNet-B0 model achieved an accuracy of 91.57%, and the ViT model achieved an accuracy of 76.97%. The proposed method outperformed baseline classification models while performing pixel-level detection.

### 3.4. ILD Interval Change Classification Accuracy

The ability of the model to identify changes in ILD status over different time intervals was assessed using follow-up CXR images. The results are shown in Table 5. The overall classification accuracy of the model was 85.29%, indicating the accuracy of disease tracking using the extent score. The model achieved a detection accuracy for aggravation of 88.24%, a precision of 72.58%, a recall of 80.35, an F1-score of 76.27%, and a specificity of 90.66%. In cases with no interval changes on chest radiographs, the model demonstrated an accuracy of 85.29%, a precision of 93.08%, a recall of 86.04%, an F1-score of 89.42%, and a specificity of 83.33%. However, the accuracy in identifying improvement was 97.06%, with a precision of 58.82%, recall of 100%, F1-score of 74.07%, and specificity of 96.93%. The relatively low precision compared to other classes is attributed to the class imbalance caused by the small number of improvement cases.

**Figure 8:**
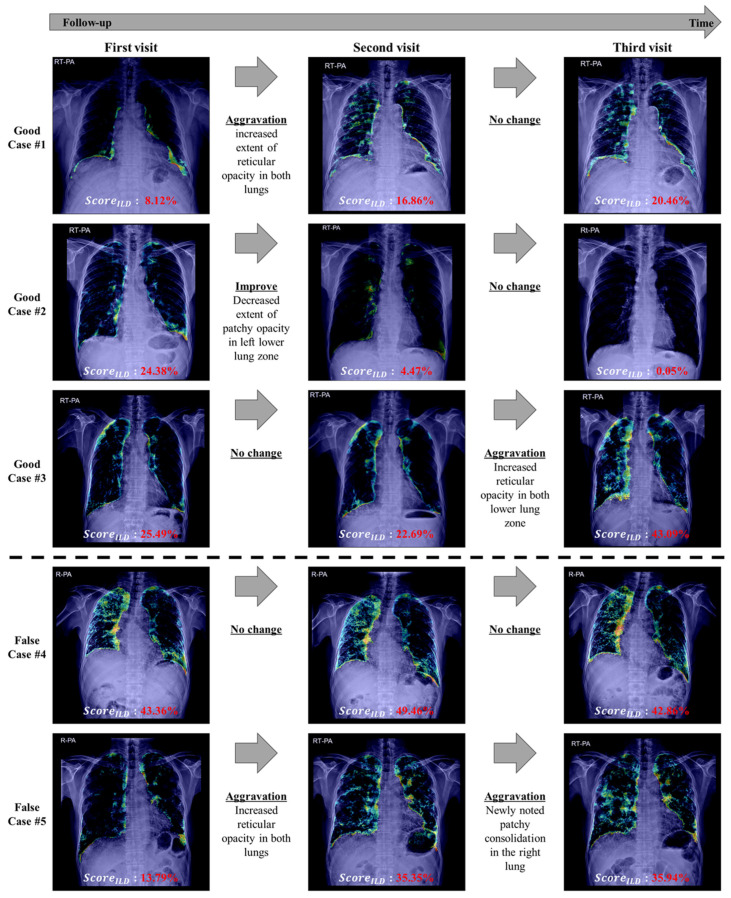
ILD interval classification results. The results using follow-up CXR images from a patient with ILD were interpreted through the model outputs (extent scores and ILD pixel-wise detection results) to show the disease progression over time. Each row represents an individual patient case; the images are arranged chronologically from left to right to represent the first, second, and third visits. The extent score displayed below each image indicates the ILD severity. The actual classification (aggravation, no change, and improvement) is shown below the directional arrows to indicate the correlation with the actual diagnostic interpretation.

Figure 8 displays the sequential results from the follow-up dataset. Across the intervals, the images illustrate the model outputs (extent score and pixel-wise detection results), assessing ILD progression (aggravation) or improvement. The pixel-wise detection results correspond with the analyzed changes in disease status alongside the class. Cases 4 and 5 in Figure 8 represent incorrectly predicted cases. For Case 4, the difference between the first and second visits was classified as no change; the extent score increased by 6.1, resulting in classification as aggravation. Conversely, although classified as no change between the second and third visits, the extent score decreased by 6.9, leading to its classification as an improvement. For Case 5, the difference between the second and third visits was labeled as aggravation; with an increase of only 0.6, it was classified as no change.

## 4. Discussion

With the development of antifibrotic drugs, the focus has been shifted toward the detection of a subset of ILD patients with progressive and irreversible fibrotic change and the determination of precise timing for the use of antifibrotic drugs [2]. The definition of PPF described in the previous guideline includes radiological evidence of disease progression, such as an increase in the extent or severity of traction bronchiectasis and new ground-glass opacity with traction bronchiectasis [2]. Thus, the detection of changes between sequential images is important for ILD patients. 

Although HRCT is reliable for ILD patients, chest radiographs are still commonly used due to their broad utility and minimal radiation exposure [5]. However, due to inbuilt limitations such as poor spatial resolution and superimposition of adjacent structures, subtle imaging features may not be visualized, making it difficult for clinicians, especially those who are not ILD specialists, to detect ILD [30,31]. According to previous studies by Hoyer et al., there is a considerable diagnostic delay in patients with IPF [32]. They reported a median diagnostic delay of 2.1 years that was mainly attributable to time from onset of symptoms in patients until first healthcare contact, time from contact with the first general practitioner until further referral, and time from the first visit to a community hospital until ILD center referral [32]. These findings indicate that it is crucial for physicians who are not ILD specialists to suspect ILDs on chest radiographs and to recommend patients to referral centers when appropriate [10,32]. Since our study shows relatively similar diagnostic accuracies to previous studies with radiologists, our model might be helpful among clinicians, especially those who are not ILD specialists; according to previous studies with radiologists of at least 10 years of experience in diagnosing ILD on chest radiographs, the overall sensitivity, specificity, positive predictive value, negative predictive value and diagnostic accuracy of chest X-ray in diagnosing ILD was 80.0%, 82.98%, 90.0%, 68.42%, and 81.02%, respectively [31].

This study introduces a novel approach to promote advances in ILD management by quantifying and visualizing ILD lesion areas on chest radiographs. Using an image-to-image translation model, our study demonstrates enhanced capabilities in pixel-wise detection of ILD areas in CXR images, with an accuracy of 92.98%. This marks a significant improvement over the traditional Grad-CAM applied ResNet-34 model. As shown in Figure 1 and Figure 7, the proposed method surpasses Grad-CAM in delineating more precise disease areas at the pixel level, enabling accurate quantification. The proposed method demonstrated an average accuracy of 85.29% in analyzing disease intervals, indicating potential use for disease monitoring. Moreover, based on our research findings, a quantitative approach for evaluating pixel-wise changes in ILD may also be helpful in reducing interobserver variance, a well-known challenge in radiological assessment. It represents an advancement in consistency in clinical diagnostics and ensures that clinical decisions are standardized and based on objective data.

Our study shows reliable accuracy in detecting disease progression in ILD patients; in clinical settings, this model may help clinicians recognize disease progression and identify the appropriate time to start treatment. However, in our study, we still observed discrepancies between the evaluations of radiologists and the results derived from the model, in some cases assessing changes in ILD. As mentioned above, this could be attributed to variations in the degree of inspiration, changes in the patient’s position, and differences in imaging equipment settings. Therefore, further research addressing these issues may be necessary.

Despite its efficacy, this study has some limitations. First, we did not assess detailed radiographic abnormalities that may mimic reticular opacities of ILD, such as emphysematous change and cystic lung disease, which may be concurrently present with underlying ILD. Second, the evaluation was conducted using a single device, raising questions concerning the generalizability of the findings. Third, the use of a predefined threshold value for the ILD extent score in interval change analysis led to some misclassifications (as shown in Figure 8, Cases 4 and 5), indicating that although our model is effective, it should be used as a complementary tool alongside clinical judgment. However, despite the instances of misclassification, the overall accuracy of the model did not diminish.

## 5. Conclusions

This study successfully demonstrated a quantitative approach for evaluating pixel-wise changes in ILD on chest radiographs, with good performance in detecting ILD and assessing interval changes in consecutive chest radiographs with quantitative scoring. The proposed method, using weakly supervised learning and image-to-image translation, offers a more detailed and objective assessment of disease changes than conventional methods. Our results show high accuracy in detecting ILD and assessing progression using the ILD extent scoring algorithm and were validated against follow-up CXR data. Therefore, the proposed method may help clinicians detect the disease progression of ILD on chest radiographs, contributing substantially to disease management and therapeutic strategy development for patients with ILD.

## Figures and Tables

**Table 1:** Application of machine learning in chest radiographs for diagnosis of ILD in previous studies. DL, deep learning; CNN, convolutional neural network; ILD, interstitial lung disease.

	Clinical Application	Machine Learning Method
**Park et al.** [6]	Feasibility of DL-based detection system for multiclass lesions (nodule/mass, interstitial opacity, pleural effusion, and pneumothorax)	Multitask CNN
**Namet al.** [7]	DL algorithm detecting 10 common abnormalities (pneumothorax, mediastinal widening, pneumoperitoneum, nodule/mass, consolidation, pleural effusion, linear atelectasis, fibrosis, calcification, and cardiomegaly)	ResNet34-based deep CNN
**Sung et al.** [8]	Comparison of observer performance in detecting and localizing major abnormal findings (nodules, consolidation, interstitial opacity, pleural effusion, and pneumothorax) with/without DL-based detection system	DL algorithm (VUNO Med-Chest X-ray, version 1.0.0)
**Kim et al.** [9]	Evaluation of the utility of a DL algorithm for detection of reticular opacity on chest radiographs of patients with surgically confirmed ILD	DL algorithm (VUNO Med-Chest X-ray, version 1.0.0)
**Nishikiori et al.** [10]	DL algorithm to detect chronic fibrosing-ILDs	DenseNet-based Deep CNN

**Table 2:** Characteristics of training and evaluation CXR datasets. The table presents data from both the training and evaluation datasets used in the study. Each dataset column includes the number of cases, distribution by sex (sex, F/M), and age statistics (age, mean ± standard deviation).

	Training Dataset		Evaluation Dataset	
	ILD	Normal	ILD (F/U)	Normal
Number of cases	5266	5266	397	500
Sex (F/M)	2284/2982	3264/2002	183/214	257/243
Age (mean ± std)	65.5 ± 10.4	52.7 ± 15.6	64.1 ± 8.5	52.9 ± 15.7

**Table 3:** Evaluation of CUT model image-to-image translation fidelity performance. This table presents the performance evaluation of the CycleGAN and contrastive unpaired translation (CUT) model across two datasets: normal and ILD. The performance metrics include SSIM and PSNR, shown as values for each dataset.

Model		CycleGAN		CUT	
Evaluation metrics		SSIM	PSNR	SSIM	PSNR
Category of dataset	Normal	0.88	23.68	**0.97**	**36.43**
	ILD	0.71	18.46	**0.90**	**26.61**

**Table 4:** ILD classification results. This table details the classification performance of the baseline and proposed models for identifying ILD. The evaluated models include classification models using VGG16, ResNet-34, EfficientNet-B0, ViT, probabilistic Grad-CAM, and image translation models using CycleGAN and CUT. The performance metrics shown are accuracy, precision, recall, and F1-score.

Task	Model	Accuracy	Precision	Recall	F1-Score
Classification model	VGG16	68.65%	72.82%	71.19%	68.45%
	ResNet-34	92.11%	92.03%	91.84%	91.93%
	EfficientNet-B0	91.57%	91.89	90.93%	91.30%
	ViT	76.97%	76.62%	77.05%	76.72%
Abnormal area detection	Probabilistic Grad-CAM [16]	84.76%	85.65%	83.40%	84.02%
Image-to-Image translation model	CycleGAN [18] with extent scoring algorithm	81.20%	73.14%	89.16%	76.96%
	CUT [21] with extent scoring algorithm	92.98%	98.54%	85.13%	95.68%

**Table 5:** Interval change classification performance. This table shows the classification performance of the model in identifying interval changes in ILD status based on follow-up datasets. Performance was analyzed by three interval ratings: aggravation, no change, and improvement. The performance of each class is expressed as a percentage using accuracy, precision, recall, F1-score, and specificity.

Interval Class	Accuracy	Precision	Recall	F1-Score	Specificity
Aggravation	88.24%	72.58%	80.35%	76.27%	90.66%
No change	85.29%	93.08%	86.04%	89.42%	83.33%
Improvement	97.06%	58.82%	100%	74.07%	96.93%
Total class	85.29%	85.29%	88.24%	85.29%	92.65%

## Data Availability

Data related to this study cannot be released due to the information security policies of the hospitals.
